# Cold Tolerance of Photosynthetic Electron Transport System Is Enhanced in Wheat Plants Grown Under Elevated CO_2_

**DOI:** 10.3389/fpls.2018.00933

**Published:** 2018-07-04

**Authors:** Xiancan Zhu, Shengqun Liu, Luying Sun, Fengbin Song, Fulai Liu, Xiangnan Li

**Affiliations:** ^1^Northeast Institute of Geography and Agroecology, Chinese Academy of Sciences, Changchun, China; ^2^Department of Plant and Environmental Sciences, Faculty of Science, University of Copenhagen, Copenhagen, Denmark

**Keywords:** cold tolerance, *Triticum aestivum*, photosynthesis, chlorophyll *a* fluorescence, CO_2_ elevation

## Abstract

The effects of CO_2_ elevation on sensitivity of photosynthetic electron transport system of wheat in relation to low temperature stress are unclear. The performance of photosynthetic electron transport system and antioxidant system in chloroplasts was investigated in a temperature sensitive wheat cultivar Lianmai6 grown under the combination of low temperature (2 days at 2/−1°C in the day/night) and CO_2_ elevation (800 μmol l^−1^). It was found that CO_2_ elevation increased the efficiency of photosynthetic electron transport in wheat exposed to low temperature stress, which was related to the enhanced maximum quantum yield for electron transport beyond Q_A_ and the increased quantum yield for reduction of end electron acceptors at the PSI acceptor side in plants under elevated CO_2_. Also, under low temperature, the activities of ATPases, ascorbate peroxidase, and catalase in chloroplasts were enhanced in wheat under elevated CO_2_. It suggested that the cold tolerance of photosynthetic electron transport system is enhanced by CO_2_ elevation.

## Introduction

Low temperature stress is one of the most critical environmental stimuli affecting crop plant growth and grain yield. As a temperate plant, wheat is tolerant to some lower temperatures during the vegetative stage, but it is very sensitive to low temperature stress during the reproductive stage (Whaley et al., 2004). In North Europe, spring wheat is frequently suffering from frost damage in late May and early June when the plants commence reproductive stage. Studies have shown that the low temperature during vegetative stage could cause yield loss up to 10% (Frederiks et al., 2012). The cold-induced damage to photosynthetic apparatus is the key limitation in energy supply to plant growth. Photosynthesis converts light energy into ATP and redox equivalents (NADPH), which is the primary metabolic sink for plant growth (Ruelland et al., 2009). Low temperature stress rapidly affects the photosynthesis by direct and indirect effects (Thakur and Nayyar, 2013). For instance, low temperature stress increases membrane viscosity and restricts the diffusion of plastoquinone, to inhibit the thylakoid electron transport (Thakur and Nayyar, 2013). Light energy trapping by the antenna of PSI and PSII and its contribution to drive charge-separation in the reaction centers (RCs) is easily disturbed by low temperature, due to the chlorophyll antenna complexes trap more energy than that can be processed biochemically (Ensminger et al., 2006). Under such circumstances, thylakoid membranes become over-energized. One of the consequences of this over-energized state is photodamage, primarily caused by the overproduction of reactive oxygen species (ROS) (Ashraf and Harris, 2013). Low temperature stress often associates with ROS accumulation. It has been well documented that the activities of the scavenging enzymes are lowered by low temperature stress (Li et al., 2013); hence, the scavenging systems cannot counterbalance the ROS formation that is always associated with chloroplastic electron transfer reactions (Li et al., 2014).

It has been well documented that the increasing atmospheric CO_2_ concentration (hereafter abbreviated to [CO_2_]) has both direct and indirect effects on plant growth and stress responses in wheat (Li et al., 2001; Pinter et al., 2001; Medina et al., 2016; Varga et al., 2017). For instance, elevated [CO_2_] has a direct and positive effect on the photosynthesis of wheat plants and thus stimulates plant growth along with impacts on other physiological processes including nitrogen metabolism (Stitt and Krapp, 1999; Wang et al., 2013). Almost all studies showed the mitigation effects of elevated [CO_2_] on abiotic stress in wheat (Shanmugam et al., 2013; Medina et al., 2016; Li et al., 2017).

The heat stress is considered to occur always along with [CO_2_] elevation in future climate, and many researches has focused on the combined effects of heat stress and [CO_2_] elevation on wheat (Sanchez de la Puente et al., 2000; Dias de Oliveira et al., 2013; Benlloch-Gonzalez et al., 2014; Vicente et al., 2015). The increased climatic variability also leads greater risk of the crop being exposure to the extreme low temperature stress (Mäkinen et al., 2017). However, less attention has been paid to the interaction of [CO_2_] elevation and low temperatures. In this study, the performance of the photosynthetic electron transport system in wheat plants under the combination of low temperature and [CO_2_] elevation was analyzed. It was hypothesized that the response of photosynthetic electron transport system to cold stress in wheat is affected by [CO_2_] elevation.

## Materials and Methods

### Experimental Design and Materials

A cold sensitive wheat cultivar Lianmai6 was used in a pot experiment at ambient (400 μmol l^−1^, for ambient [CO_2_] treatment) and elevated [CO_2_] (800 μmol l^−1^, for elevated [CO_2_] treatment), respectively, from September 15 to February 15 in 2015. Four seeds of wheat cv. Lianmai6 were sown in each pot, filled with 4.8 kg of sandy loam. Before filling the pots, the soil was pre-mixed with 2 g N, 1 g P_2_O, and 1.4 g K_2_O per pot, and no additional fertilization was applied in later stages.

### Treatments

From sowing, half of the plants were grown in the greenhouse cell with ambient [CO_2_] (400 ppm, a[CO_2_]), and another half were grown in the cell with elevated [CO_2_] (800 ppm, e[CO_2_]). The elevated [CO_2_] treatment was applied by emission of pure CO_2_ from a bottle tank, released in one point and distributed in the phytotrons through internal ventilation (Yan et al., 2017). The [CO_2_] in the greenhouse was monitored every six seconds by CO_2_ Transmitter Series GMT220 (Vaisala, Helsinki, Finland) during the whole growing season. At the head emerging stage (*Zadoks* 50), half of wheat plants in each cell were transferred into low temperature phytotrons for a 2-day low temperature stress treatment (2/−1°C in the day/night). The rest of wheat plants were grown under the normal temperature (26/16°C in the day/night). The other environmental condition in low temperature phytotrons was set as the same as that in normal temperature phytotrons. Therefore, four treatments were included: EN, elevated [CO_2_] + normal temperature; AN, ambient [CO_2_] + normal temperature; EL, elevated [CO_2_] + low temperature stress; and AL, ambient [CO_2_] + low temperature stress. The experiment was a randomized block design. Each treatment had four replicates, and each replicate consisted of 5 pots. The flag leaves in each replicate were collected for chloroplast isolation and measurements of ATPase and antioxidant enzyme activity, just after the chlorophyll *a* fluorescence measurement.

### Chlorophyll *a* Fluorescence

The fast chlorophyll *a* fluorescence induction curve was measured on the flag leaf just after low temperature treatment using a Plant Efficiency Analyzer (Pocket-PEA; Hansatech, Norfolk, United Kingdom). Before measuring, plants were dark adapted for 0.5 h. The collected data were processed by the program PEA Plus 1.04, and Biolyzer 3.0 software^1^ (Bioenergetics Laboratory, Geneva, Switzerland) was used to calculate the fast chlorophyll *a* fluorescence induction (OJIP) test parameters.

### Chloroplasts Isolation and ATPase Activity

Leaf samples (6 g) were ground in 30 mL of extraction buffer [0.45 M sucrose, 15 mM 3-(N-morpholino) propanesulfonic acid (MOPS), 1.5 mM ethylene glycol tetra acetic acid (EGTA), 0.6% polyvinylpyrro-lidone (PVP), 0.2% bovine serum albumine (BSA), 0.2 mM phenylmethylsulphonyl fluoride (PMSF), and 10 mM dithiothreitol (DTT)]. Homogenate was filtered through gauze (8 layers), and the filtrate was centrifuged at 2000 ×*g* for 5 min. The sedimentation was resuspended with sorbitol resuspension medium [SRM, 0.33 M sorbitol in 50 mM 4-(2-hydroxyethyl)-1-piperazineethanesulfonic acid] and layered on the top of a layered system (7 mL, 35%, 80% Percoll) for the step gradients. The chloroplasts were collected and washed with 2 mL SRM followed by centrifugation at 1100 ×*g* for 10 min. The activities of Ca^2+^- and Mg^2+^-ATPase in the chloroplasts suspension were measured following the method of Li et al. (2015b).

### H_2_O_2_ Concentration and Antioxidant Enzyme Activity

Following our previous methods, H_2_O_2_ concentration was measured by monitoring the absorbance of titanium peroxide complex at 410 nm (Zuo et al., 2017). The ascorbate peroxidase (APX) activity was determined by monitoring the decrease at 290 nm, and the activity of superoxide dismutase (SOD) was measured by monitoring the inhibition of photochemical reduction of nitroblue tetrazolium (NBT) (Zuo et al., 2017). The catalase (CAT) activity was measured as described by Li et al. (2014).

### Leaf Pigment Concentration

Fresh leaf (0.1 g) was sliced and incubated in 50 ml of pigment extraction solution containing acetone and anhydrous ethanol (1:1, v/v) in dark at 25°C for 12 h. The supernatant was collected and measurement for absorbance at 663 and 647 nm. Concentrations of total chlorophyll were then calculated according to Arnon (1949).

### Leaf ATP Concentration

The ATP was extracted following the methods of Stewart and Guinn [39] and was measured with the ATP Bioluminescent Assay Kit (Baomanbio, Shanghai, China) as described by Zheng et al. (2009).

### Statistical Analysis

All data were subjected to the one-way ANOVA to determine the significant differences between treatments using the software of SPSS (Ver. 19.0 SPSS, Chicago, IL, United States). Energy pipeline leaf model of phenomenological fluxes (per cross-section, CS) was performed using the Biolyzer 3.0 software (Bioenergetics Laboratory, Geneva, Switzerland).

## Results

### Chl *a* Fluorescence Transient

**FIGURE 1:**
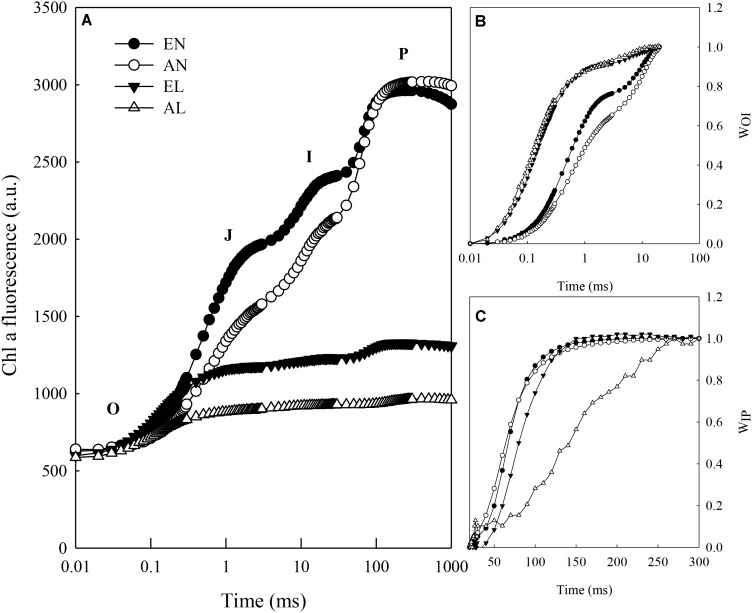
Chlorophyll *a* fluorescence transient of dark adapted leaves exposed to combination of cold stress and elevated CO_2_ in wheat. **(A)** Fluorescence intensity on logarithmic time scale; **(B)** Variable fluorescence *F*_t_–*F*_o_ to the amplitude *F*_I_–*F*_o_; **(C)** Ratio of variable fluorescence *F*_t_–*F*_I_ to the amplitude *F*_P_–*F*_I_. Abbreviations of treatments are explained as followed: EN, elevated CO_2_ + normal temperature; AN, ambient CO_2_ + normal temperature; EL, elevated CO_2_ + low temperature stress; AL, ambient CO_2_ + low temperature stress.

The increase in leaf fluorescence transients in wheat plants under normal temperature treatments (EN and AN) showed a typical OJIP shape (**Figure 1A**). Under low temperature treatments (EL and AL), it showed repressed fluorescence transients, particularly at step I (30 ms) and P (100 ms). The main changes of fluorescence transients were normalized from O to step I and step I to step P and presented as relative variable fluorescence W_OI_ and W_IP_, respectively (**Figures 1B,C**). Significant changes in W_OI_ were found between normal temperature treatments and low temperature treatments, which were due to the reductions between PSI and reduced NADP^+^. Obvious changes in W_IP_ during the fast rise period were observed under EN, AN, and EL, in relation to AL.

**FIGURE 2:**
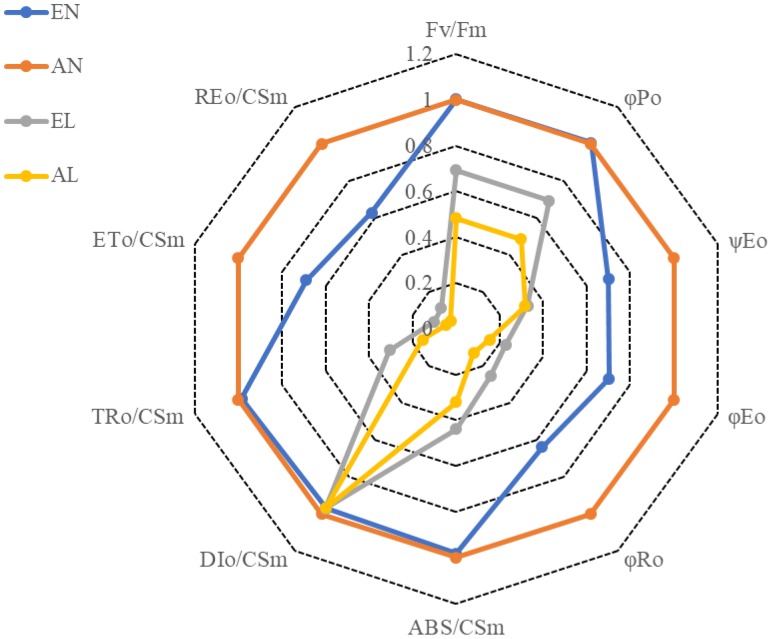
Fluorescence transient Chl *a* parameters deduced from analysis of the JIP-test of wheat as affected by cold stress and elevated CO_2_. Abbreviations of treatments are explained in **Figure 1**. Explanations of these parameters are shown in **Table 1**.

The maximum quantum yield of the PSII (Fv/Fm) were significantly reduced by a 2-day low temperature stress, compared to the normal temperature control (AN); however, the reduction in Fv/Fm was more pronounced in AL than in EL (**Figure 2**). The interpretations of the selected OJIP test parameters from the Chl a fluorescence rise transients in different treatments are shown in **Table 1**. No significant difference in the maximum quantum yield for primary photochemistry (φ_PO_) was found between EN and AN, while the φ_PO_ in EL was significantly higher than that in AL. However, there was no significant difference in quantum yield for electron transport (ET) (φ_EO_) between EL and AL. The highest value of probability that an electron moves further than Q_A_ (ψ_EO_) and quantum yield for reduction of end electron acceptors at the PSI acceptor side (RE) (φ_RO_) were found in AN, followed by EN and EL, and the lowest value was in AL.

**Table 1:** Explanations of selected JIP-test parameters used in the present study.

Fluorescence parameters	
*F*_O_	Minimal fluorescence, when all PSII RCs (reaction centers) open
*F*_V_ = *F*_t_−*F*_O_	Variable fluorescence at time *t*
*F_M_*	Maximal recorded fluorescence intensity
*W*_OI_ = (*F*_t_−*F*_O_)/(*F*_I_−*F*_O_)	Ratio of variable fluorescence *F*_t_−*F*_O_ to the amplitude *F*_I_−*F*_O_
*W*_IP_ = (*F*_t_−*F*_I_)/(*F*_P_−*F*_I_)	Ratio of variable fluorescence *F*_t_−*F*_I_ to the amplitude *F*_P_−*F*_I_
φ_PO_	Maximum quantum yield for primary photochemistry
φ_EO_	Quantum yield for electron transport (ET)
φ_RO_	Quantum yield for reduction of end electron acceptors at the PSI acceptor side (RE)
ψ_EO_	Probability that an electron moves further than Q_A_
TR_O_/CS_O_	Trapped energy flux per CS
ET_O_/CS_O_	Electron transport flux per CS
RE_O_/CS_O_	PSI acceptor per CS
ABS/CS_O_	Absorption flux per CS
DI_O_/CS_O_	Non-photochemical quenching per CS

*Subscript “o” indicates that the parameters refer to illumination onset, when all reaction centers are assumed to be open.*

**FIGURE 3:**
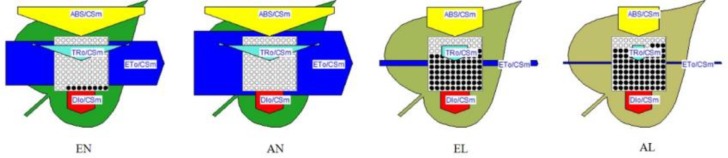
Energy pipeline leaf model of phenomenological fluxes (per cross-section, CS) of the flag leaf in wheat as affected by cold stress and elevated CO_2_. Abbreviations of treatments are explained in **Figure 1**.

The derived parameters from the OJIP curves were summarized by means of energy pipeline leaf model of phenomenological fluxes (per CS) as shown in **Figure 3**. The energy absorbed per excited cross-section (CSm) (ABS/CSm) in EL was significantly higher than AL. No significant difference in non-photochemical quenching per CSm (Dio/CSm) was observed among these four treatments. The trapped energy flux per CSm (TR_O_/CSm) was similar in EN and AN, whereas it was higher in EL than AL. The PSI acceptor per CS (RE_O_/CSm) and electron transport flux per CS (ETo/CSm) was significantly higher in EL, in relation to that in AL. In addition, these two parameters were significantly higher in EN than that in AN. The number of active RCs in PS II CS (shown by open circles in **Figure 3**) was significantly lower in the plants under low temperature stress, compared with the normal temperature control. It should be noted that the number of active RCs was higher in EL plants than AL plants.

### ROS Production and Antioxidant System

**FIGURE 4:**
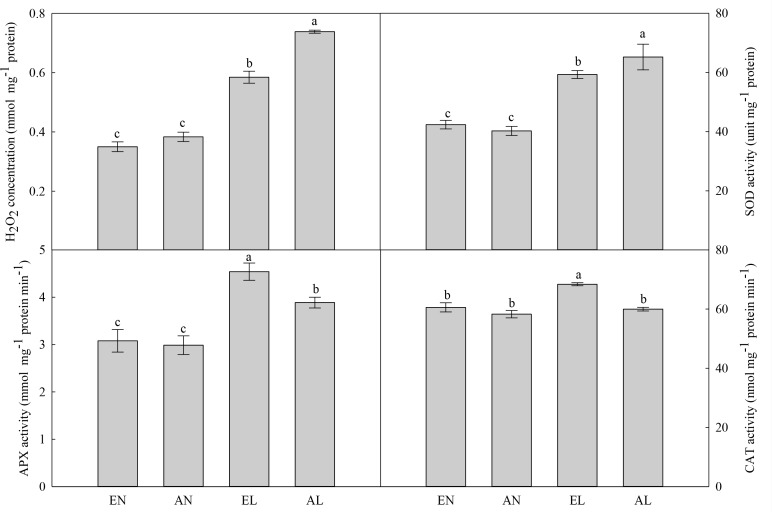
Concentration of H_2_O_2_ and activities of SOD, APX, and CAT in the flag leaf in wheat as affected by cold stress and elevated CO_2_. Abbreviations of treatments are explained in **Figure 1**. Different small letters mean significant difference at *P* < 0.05 level.

The concentration of H_2_O_2_ in the chloroplasts in flag leaf was significantly increased by AL, compared with AN (**Figure 4**). No significant difference was found in leaf H_2_O_2_ concentration between elevated [CO_2_] treatment (EN) and ambient [CO_2_] control (AN). The EL plants had remarkably lower H_2_O_2_ level in chloroplasts compared with AL plants. There was no significant difference in the activities of SOD, APX, and CAT between EN and AN. Interestingly, the SOD activity was significantly higher, while the activities of APX and CAT were lower in AL, in relation to EL.

### ATPase Activities, Total Chlorophyll, and ATP Concentrations

**FIGURE 5:**
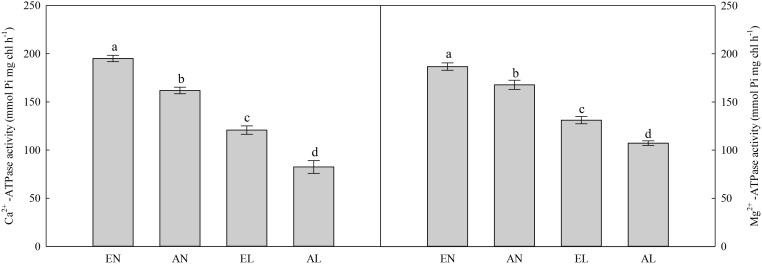
Activities of Ca^2+^-ATPase and Mg^2+^-ATPase in the flag leaf in wheat as affected by cold stress and elevated CO_2_. Abbreviations of treatments are explained in **Figure 1**. Different small letters mean significant difference at *P* < 0.05 level.

**FIGURE 6:**
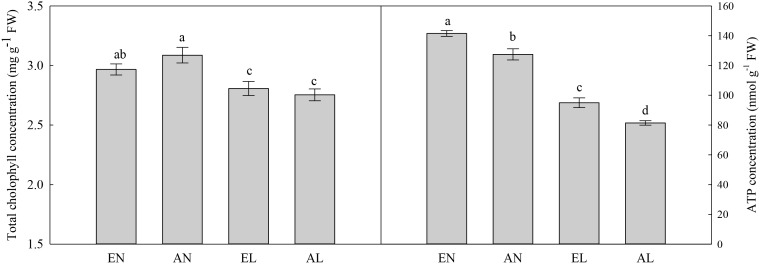
Concentrations of chlorophyll and ATP in the flag leaf in wheat as affected by cold stress and elevated CO_2_. Abbreviations of treatments are explained in **Figure 1**. Different small letters mean significant difference at *P* < 0.05 level.

In chloroplasts, the highest activities of Mg^2+^-ATPase and Ca^2+^-ATPase were in EN plants, followed by AN and EL, and the lowest values were found in AL (**Figure 5**). A similar trend was also found in ATP concentration among these treatments (**Figure 6**). In addition, the total chlorophyll concentration in flag leaf was the highest in AN, while it was the lowest in EL and AL.

## Discussion

Photosynthesis is a highly sensitive process to temperature fluctuations, since it is important to keep the balance between light energy absorbed by photosystems and energy consumed by metabolic sinks (Krček et al., 2008; Brestic et al., 2012; Zivcak et al., 2013). It has been reported that low temperatures exacerbate the imbalance between absorbed light energy and the metabolic sink, thus requiring adjustments of photosynthesis to maintain the balance of energy flow (Ensminger et al., 2006). Low temperature stress depresses photosynthesis, mainly due to its negative effects on photosynthetic electron transport system (Li et al., 2014). The fast chlorophyll *a* fluorescence induction curve has been widely used to investigate the photosynthetic electron transport as influenced by abiotic stress, such as low temperature (Strasser, 1987, 1992; Strauss et al., 2006). The present study showed repressed fluorescence transients after a 2-day low temperature stress in wheat grown under both ambient and elevated [CO_2_]. The I-P phase of the transient fluorescence kinetics indicates the changes in the electron flux from PQH_2_ to the final electron acceptor and the size of the final electron acceptor pool of PS I (Yusuf et al., 2010). Under ambient [CO_2_], the low temperature stress significantly reduced the electron flux from PQH_2_ to the final electron acceptor, compared with the normal temperature control. However, the electron flux from PQH_2_ to the final electron acceptor was similar in low temperature stressed and non-stressed plants under elevated [CO_2_]. This indicated that elevated [CO_2_] had a mitigation effect on the damage of low temperature to the electron flux from PQH_2_ to the final electron acceptor and the sink size of the final electron acceptor of PS I. The O-I part of the kinetics was affected by low temperature and elevated [CO_2_], which reveals changes in the process involving exciton capture to PQ reduction (Yusuf et al., 2010). However, the plants under elevated [CO_2_] showed a faster rise in fluorescence transient from O to I when exposed to low temperature, compared with the plants under ambient [CO_2_], indicating the process involving exciton capture to PQ reduction had a higher efficiency in plants under elevated [CO_2_].

In the present study, the wheat plants under elevated [CO_2_] possessed higher Fv/Fm than the plants under ambient [CO_2_] when exposed to low temperature, indicating that the [CO_2_] elevation favors the quantum yield of the PSII under low temperature stress. The efficiency of the light reactions, which is expressed as φ_Po_, was observed also higher in the EL plants exposed to low temperature, in relation to AL plants (Chen et al., 2011). When exposed to low temperature, the efficiency balance of the dark reactions after Q_A_, which is reflected by ψ_Eo_, was similar in plants grown under ambient and elevated [CO_2_]. The φ_Eo_ reveals the maximum quantum yield for electron transport beyond Q_A_, and it also indicates the maximum quantum yield of primary photochemistry; while φ_Ro_, represents the quantum yield for reduction of end electron acceptors at the PSI acceptor side (RE) (Chen et al., 2011). Here, these two parameters were significantly higher under low temperature stress in wheat grown under ambient and elevated [CO_2_]. It indicates that [CO_2_] elevation mitigated the damage to primary photochemistry reaction and protected the end acceptors at the PSI electron acceptor side as well as the activity of ferredoxin-NADP^+^ reductase (FNR) in wheat exposed to low temperature stress.

Based on the derived parameters from the chlorophyll *a* fluorescence induction curve, the leaf model of phenomenological energy fluxes (per CS) was used to show the changes of electron transport process under low temperature and elevated [CO_2_]. The ETo/CSm presented the reoxidation of reduced Q_A_ via electron transport over a CS of active and inactive RCs (Mehta et al., 2010). When exposed to the low temperature stress, the plants grown under elevated [CO_2_] showed a higher electron transport efficiency in CS than the plants under ambient [CO_2_]. The density of active RCs in PS II CS was also higher in the EL plants, compared with AL plants, which implies that less active RCs were converted into inactive RCs due to [CO_2_] elevation. It should be noted that the energy absorbed per excited CS (ABS/CSo) was higher in EL plants than that in AL plants, while non-photochemical quenching (DIo/CSo) was similar in both, indicating that the energy absorption efficiency of PS II was higher in plants grown under elevated [CO_2_]; however, the non-photochemical quenching in plants grown under elevated [CO_2_] was not the main way to maintain the thermo-stability of PSII in exposure to low temperature stress.

Photosynthesis in chloroplasts is one of the main sources of ROS production in plants under abiotic stress (Li et al., 2015a). As a non-radical form of ROS, H_2_O_2_ inactivates the enzymes related to photosynthesis, hence reduces the photosynthetic carbon assimilation (Li et al., 2014). Here, the cold-induced increase of H_2_O_2_ concentration was lower in EL plants as compared to AL plants. This was due to the higher activities of APX and CAT. Both antioxidant enzymes can decompose H_2_O_2_ into H_2_O and O_2_, which are important for scavenging of ROS in plants under abiotic stress (Keunen et al., 2013). The wheat plants grown under elevated [CO_2_] possessed higher ROS scavenging capacity than that under ambient [CO_2_], which benefited for the protection of photosynthetic electron transport system.

Photosynthetic electron transport generates ATP and reduces NADPH to support carbon reduction and ensure effective energy flow for plant growth (Ye et al., 2013). In consistent with the enhanced photosynthetic electron transport efficiency in plants grown under elevated [CO_2_], the ATP production was higher in EL plants compared with AL plants. This was also due to the increased activities of Mg^2+^-ATPase and Ca^2+^-ATPase in EL plants under low temperature stress, in relation to AL plants.

## Conclusion

When exposed to low temperature stress, the wheat grown under elevated [CO_2_] had a higher efficiency in photosynthetic electron transport than wheat under ambient [CO_2_]. This was due to the enhanced maximum quantum yield for electron transport beyond Q_A_ and the increased quantum yield for reduction of end electron acceptors at the PSI acceptor side (RE) in plants under elevated [CO_2_], compared with that under ambient [CO_2_]. In addition, the elevated [CO_2_] mitigated the negative effects of low temperature stress on ATP synthesis by enhancing the ATPase activities. The cold tolerance of photosynthetic electron transport system is enhanced in wheat grown under elevated [CO_2_].

## Author Contributions

XL and FL designed the experiments. XZ and XL performed the experiments. LS, SL, FS, FL, and XL wrote and revised the manuscript.

## Conflict of Interest Statement

The authors declare that the research was conducted in the absence of any commercial or financial relationships that could be construed as a potential conflict of interest.

## Footnotes

1 http://www.fluoromatics.com/biolyzer_software-1.php
